# Medical Exposure from Diagnostic Examinations and Procedures of the Swiss Population: 2023 Review

**DOI:** 10.1097/HP.0000000000002133

**Published:** 2026-03-06

**Authors:** Eleni Theano Samara, Barbara Ott, Reto Treier, Philipp R. Trueb, Florentin Krämer, Tino Schönleitner, Achim Escher, Valerie Treyer, Thomas Krause, Dorothea Dagassan-Berndt, Elmar M. Merkle

**Affiliations:** 1 Radiation Protection Unit, Zurich University Hospital, University of Zurich, Zurich, Switzerland; 2 Radiation Protection Division, Federal Office of Public Health FOPH, Bern, Switzerland; 3 BSS Economic Consultants, Basel, Switzerland; 4 Escher Consulting GMBH, Basel, Switzerland; 5 Department of Nuclear Medicine, Zurich University Hospital, University of Zurich, Zurich, Switzerland; 6 Department of Nuclear Medicine, Bern University Hospital, University of Bern, Bern, Switzerland; 7 Dental Imaging, University Center for Dental Medicine Basel UZB, University of Basel, Basel, Switzerland; 8 Department of Radiology, University Hospital Basel, University of Basel, Basel, Switzerland.

**Keywords:** computed tomography, diagnostic radiology, dose assessment, medical radiation

## Abstract

This survey aimed to estimate the radiation exposure of the Swiss population from medical diagnostic procedures performed in 2023. The survey was based on billing data and administratively collected data for radiology and nuclear medicine and on data collection for the frequency of dental examinations. A total of 12.9 million radiological examinations were performed, corresponding to 1,443 examinations per 1,000 inhabitants. The collective effective dose was 1.69 mSv per capita, presenting a 13% increase compared with the dose estimated in 2018. Despite accounting for only 14.4% of the total number of examinations, computed tomography accounted for 75.7% of the total dose. In 2023, 207 CT scans per 1,000 inhabitants were recorded, with an average dose of 1.28 mSv per capita. The highest contribution to the examination frequency (46.5%) was recorded in dentistry, but this accounted for only 0.6% of the total dose (0.01 mSv per capita). The survey resulted in an update of the national data on population dose exposure from medical diagnostic procedures.

## INTRODUCTION

Diagnostic examinations and interventional radiology procedures represent the highest contribution of manmade exposure to ionizing radiation. The average dose to the population from medical exposure was estimated to be approximately 1.7 mSv y^-1^ in high-income level countries by the last UNSCEAR worldwide survey (UNSCEAR 2022). Legislation in Switzerland requires the assessment of the development of exposure frequency and dose per exposure over time at regular intervals (Council 2017). The first such survey in Switzerland was performed in the late 1950s, and since 1998, surveys have been carried out every 5 y (Aroua et al. 2002, 2007; Le Coultre et al. 2013; Samara et al. 2012; Viry et al. 2021).

During the optimization process, various radiological protection quantities are evaluated based on expected individual and effective dose assessments. According to the International Commission on Radiological Protection (ICRP 2007), to determine the collective effective dose from radiological examinations, the frequency of the different types of examinations and procedures and the typical effective dose for each type of procedure must be estimated. The determination of the frequency of examinations can be made either by national surveys covering a well-defined sample of hospitals and medical practices or using administrative data, tariff-based billing data, or a combination of all. Different methods to determine the effective dose of the examinations are also available.

The aim of this work was to present the methodology to determine the collective effective dose for the Swiss population from medical radiological procedures and to present the results for 2023.

## MATERIALS AND METHODS

### Calculation of collective effective dose

The collective effective dose (*S*) can be calculated by the following formula:


$$S=\;\underset{i}{\sum }\;E_{i}\cdot N_{i},$$ (1)

where *E*_*i*_ is the effective dose vector (E) for a subgroup *i* and *N*_*i*_ is the number of individuals in the subgroup during the period to be considered (ICRP 2007). The period in this survey was the entire year 2023.

Finally, the collective effective dose per capita is derived by calculating the ratio of *S* to the population size. In 2023, the population of Switzerland that was classified as permanent residents was 8,962,258 (Federal Statistical Office 2024).

### Frequency vector (number of examinations during the considered period)

The estimate of the number of examinations performed is based on billing and administrative data supplemented by primary data collected specifically for this survey.

**Fig. 1:**
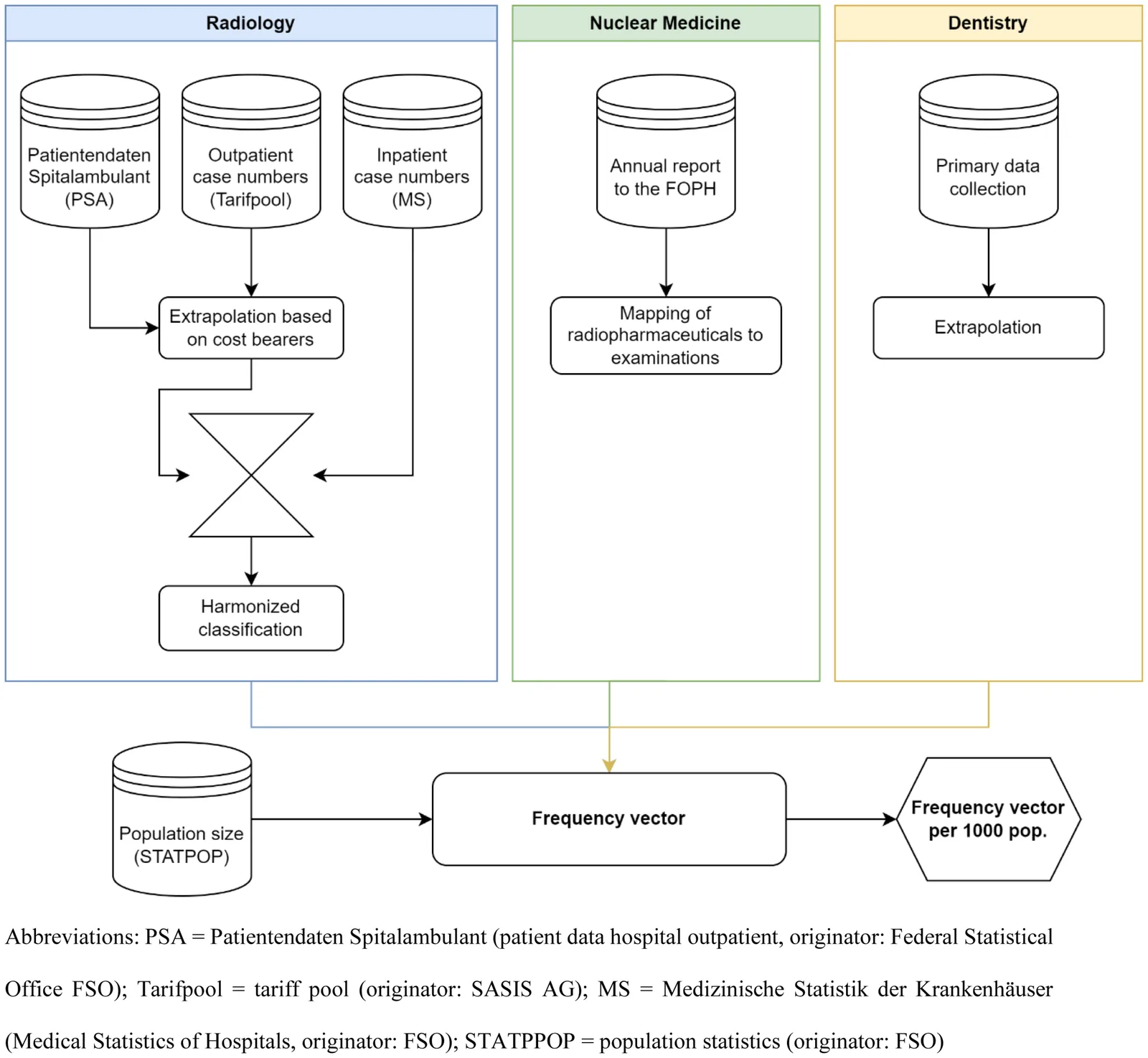
Diagram for the calculation of frequencies of all modalities. Abbreviations: PSA = Patientendaten Spitalambulant (patient data hospital outpatient, originator: Federal Statistical Office FSO); Tarifpool = tariff pool (originator: SASIS AG); MS = Medizinische Statistik der Krankenhäuser (Medical Statistics of Hospitals, originator: FSO); STATPPOP = population statistics (originator: FSO).

For radiology, the frequencies for outpatients were derived from billing data (TARMED) and from procedure codes (CHOP) for stationary care (Fig. 1). The codes from these systems were harmonized into a common classification of modalities, anatomical regions, and examination protocols. This facilitated the aggregation of data from out- and inpatients. The examinations were identified according to the relevant codes and counted. The two tariff systems (TARMED and CHOP) had different resolutions. For example, diagnostic mammograms are coded more granularly in CHOP than in TARMED, while the opposite was true for conventional x rays of the extremities. To finally add up the frequencies from the two tariff systems, we therefore apply a harmonized classification. This was strongly based on the two previous surveys (Bize et al. 2018; Le Coultre et al. 2013) and on the healthcare atlas of the Swiss Health Observatory (Swiss Health Observatory 2024).

The CHOP codes for stationary patients represent a complete survey. For outpatient care, the frequencies were based on two invoice systems; one covering outpatients treated in hospitals (PSA) and one covering patients treated by other healthcare providers (tariff pool). The tariff pool only contained invoices that were billed via statutory health insurance (OKP); services provided by accident insurance (UV), disability insurance (IV), and military insurance (MV), as well as services paid out-of-pocket or via supplementary insurance, were therefore not included in the tariff pool. Since the PSA records the billing of all cost bearers, this data source was used to determine the ratio between the number of examinations billed via statutory health insurance and the number of examinations billed via other cost bearers. Subsequently, we used the ratio from the hospital outpatient sector to extrapolate the outpatient frequencies of the tariff pool. This step was performed before adding the outpatient and inpatient frequencies.

The approach to record frequencies in interventional radiology was essentially the same as in conventional radiology. The extraction, counting, and aggregation of examination frequencies in interventional radiology presented additional challenges:

i) Different granularity: While interventional examinations are coded very granularly in the CHOP, TARMED partly follows a so-called “catch all” principle. For example, one single code was used to bill a wide variety of therapeutic and diagnostic interventions under fluoroscopy (n = 32,663 in 2023). This made it impossible to meaningfully differentiate between the examinations in terms of dose and thus also to transfer data between the outpatient and inpatient sectors.
ii) Identification of anchor codes: An interventional procedure performed in the inpatient sector usually consists of several CHOP codes. The challenge was to define the so-called anchor codes that were always billed and could therefore be counted to record the frequency of examinations. Due to these challenges, a pragmatic approach was taken: only the frequencies for interventions that could be clearly distinguished in the data were recorded.

Regarding nuclear medicine examinations, the annual report to the Federal Office of Public Health (FOPH) was analyzed. This report must be submitted by all healthcare providers to the FOPH every year and includes all outpatient and inpatient sectors. The data on the number of patients and the activity applied per year (MBq) were further broken down by individual radiopharmaceuticals. To calculate the frequency of radiation-exposing examinations in nuclear medicine, first, the radiopharmaceuticals were assigned to uniform categories, as the raw data contained different spellings. Second, the radiopharmaceuticals were mapped to the higher-level modalities PET and SPECT as well as to the individual anatomical regions/protocols. The scintigraphies were counted as SPECT, as the main difference between the two is the imaging technique and therefore no meaningful differentiation was possible based on the radiopharmaceuticals. As a final step, the total number of patients (= number of sessions) was calculated for each anatomical region/protocol as described in the previous step.

A PET examination is always accompanied by a native CT scan. In addition, a contrast agent is often added to examine the affected anatomical site more closely. In SPECT, accompanying CT scans (usually without contrast agent) are occasionally performed. The CT scans did not affect the frequency of examinations, as it was already accounted for the PET/SPECT scans.

For dental radiology, the frequencies were collected via a dedicated online survey with structured questionnaires allowing interval-based reporting, converted to annual counts. All dental device license holders were contacted (a list of addresses was provided by the Federal Office of Public Health 2024). It covered a total of 3,631 dental practices and departments.

Frequencies are calculated as the number of examinations per 1,000 permanent individuals, with adjustments made for data completeness and coding changes over time. Age and gender analysis was also possible using the billing data but not for nuclear and dental examinations.

### Dose vector

The effective dose for each type of examination was estimated using different methods: (1) dosimetric data collected during surveys for the determination of diagnostic reference levels, (2) dosimetric data collected by the FOPH in the framework of this study (Ott et al. 2025), (3) literature review, (4) data from a dose management system (DMS) of one university hospital, and (5) older national and international surveys. The detailed methodology and calculations of the dose vector can be found in a separate article.^9^

## RESULTS

In total, 12.9 million radiological examinations were performed in Switzerland in 2023 with an associated collective effective dose of 15,220.5 person-Sv. This results in an average collective effective dose of 1.69 mSv and 1.4 examinations per capita. Table 1 summarizes the distribution of frequencies (number of examinations in thousands, frequency per 1,000 inhabitants), and doses (collective effective dose and effective dose per capita) for all radiological modalities, including nuclear medicine examinations.

**Table 1.** Frequency and dose data for the Swiss population exposure to diagnostic examinations and procedures in 2023.

Modality	Frequency of examinations (in thousands)	S (person-Sv)	Frequency of examinations per 1,000 population	E per capita (mSv)
*Radiology*				
CT	1,859	11,492.2	207.4	1.28
Radiography	4,012	355.1	447.7	0.04
Mammography	438	270.1	48.9	0.03
Fluoroscopy	109	245.3	12. 2	0.03
Interventional radiology - diagnostic procedures	103	711.0	11.5	0.08
Interventional radiology - therapeutic Procederes	114	1,023.3	12.7	0.12
DEXA	139	0.1	15.5	0.00002
*Dental medicine*				
Dental radiology	6,015	92.6	671.1	0.01
*Nuclear medicine*				
SPECT/CT - Scintigraphy	57	276.0	6,323	0.03
PET/CT	84	754.7	9.33	0.08
***Total***	***12,929.5***	***15,220.5***	***1,442.7***	***1.69***

**Fig. 2:**
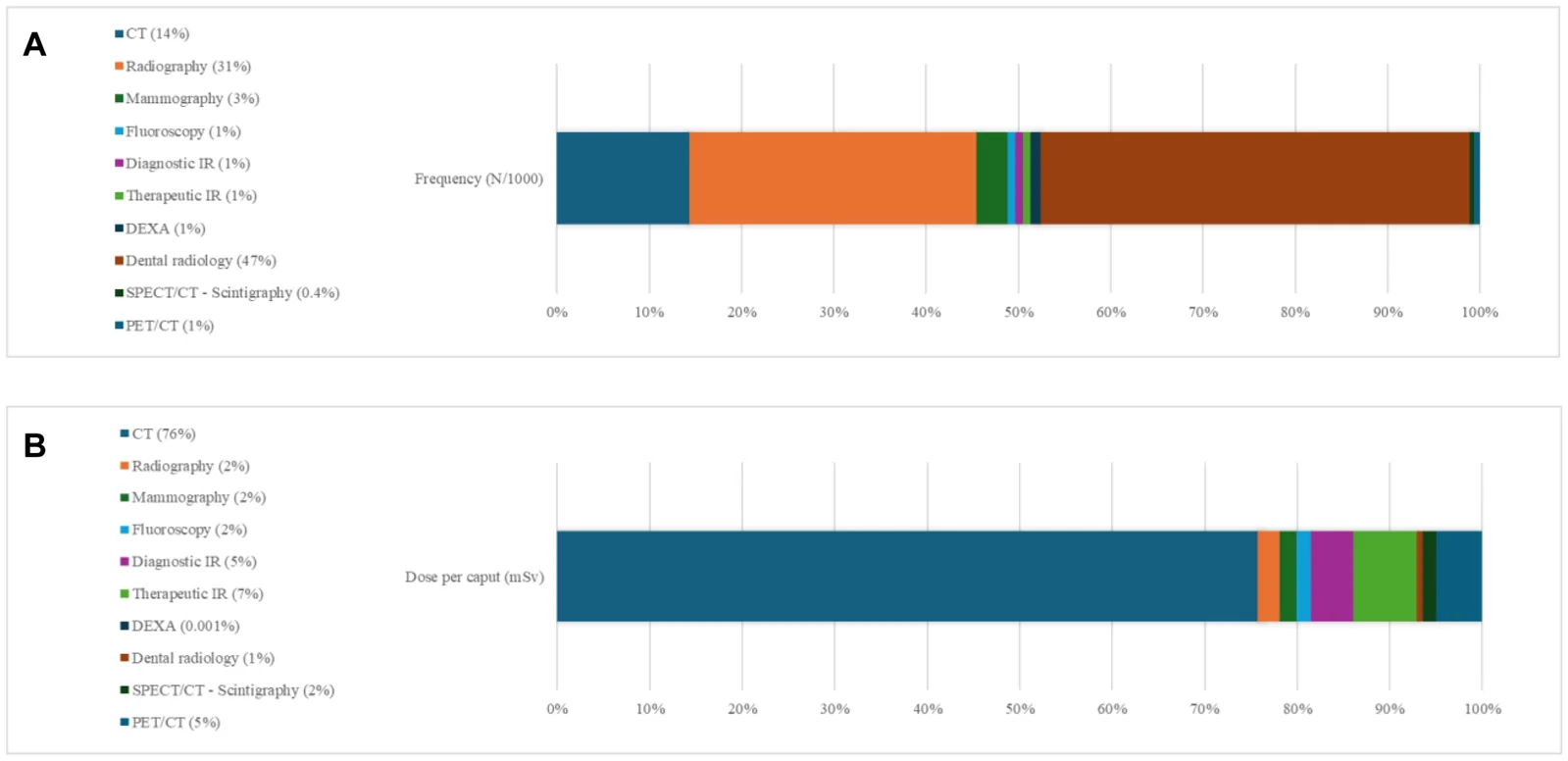
(a) Shows the frequency contributions of the various modalities, (b) Shows the dose contributions of the various modalities.

Fig. 2a shows the contribution of each modality to the total number of examinations, while Fig. 2b shows their contribution to the collective effective dose. The highest contributions to the total number of examinations come from dental radiology (47%) and radiography (31%), followed by CT, which contributed 14%. In terms of effective dose per capita, the contribution of CT was the highest with 76%, followed by diagnostic and therapeutic interventional procedures (11.5%) and PET/CT examinations (5%).

### Radiology

Table 2 presents the results for computed tomography (CT) scans. A total of around 1.9 million CT scans were performed in 2023, excluding the planning CT for radiation therapy treatments. The frequency was 207 per 1,000 inhabitants, and the corresponding effective dose per capita was 1.28 mSv. The CT scans of the abdomen, spine, and thorax accounted for around 62% of the frequency and 76% of the dose. The effective CT dose per capita was limited to a few dedicated anatomical regions. The effective dose per examination also showed a heterogeneous picture, ranging from 0.06 mSv (ankle/foot) to 11.4 mSv (spine).

**Table 2.** The table shows the effective dose, collective effective dose, frequency per 1,000 inhabitants, and dose per capita for CT examinations.

CT examination per anatomical region	E per examination(mSv)	S(person-Sv)	Frequency of examinations per 1,000 inhabitants	E per capita (mSv)
Abdomen	9.9	481.14	48.6	0.481
Spine	11.4	316.92	27.8	0.317
Thorax	3.5	180.25	51.5	0.180
Brain	2.4	60.96	25.4	0.061
CT Intervention (all anatomical regions)	8	52.8	6.6	0.053
Pelvis/Sacroiliac joint	7	39.9	5.7	0.040
Hip/Thigh	9.3	38.13	4.1	0.038
Upper abdomen	11.4	36.48	3.2	0.037
Neurocranium	2.1	19.53	9.3	0.020
Neck	2.1	18.9	9	0.019
Shoulder/Upper arm	5.8	12.76	2.2	0.013
Knee/Lower leg	2.7	12.96	4.8	0.013
Wrist/Hand	1.9	6.08	3.2	0.006
Elbow/Lower arm	3.2	3.2	1	0.003
Others	5	2	0.4	0.002
Ankle/Foot	0.06	0.276	4.6	0.0003

A total of just over 4 million x rays were performed in 2023, corresponding to around 448 examinations per 1,000 inhabitants. The effective dose per capita was 0.04 mSv. The most common x-ray examination remains the thorax radiography, accounting for 17% of all x rays. While x rays of the extremities (arm, hand, leg, foot, etc.) accounted for just under 60% of all x rays, they contribute only 0.7% to the dose. Conversely, the frequency contribution of radiography of spine and pelvis/hip is slightly over 12%, but the dose contribution is 47%.

In 2023, around 438 thousand mammographies were performed, resulting in 30 diagnostic and 19 screening mammographies. The effective dose per capita is 0.03 mSv, with just under 65% coming from diagnostics.

A total of just under 139,000 DXA scans were recorded in 2023, with a frequency of 15.5 per 1,000 inhabitants. However, the contribution to the total dose is close to 0%.

In 2023, around 110,000 conventional fluoroscopy examinations were performed (12.2 per 1,000 inhabitants) leading to an effective dose of 0.027 mSv per capita. The most common were functional fluoroscopy examinations of joints, thorax, and spine, followed by cystography, gastrointestinal diagnostics (e.g., pharyngoesophageal imaging), and arthrography. Functional fluoroscopy accounted for around 50% of the dose.

The results for diagnostic and therapeutic interventional examinations are presented in Table 3. In 2023, a total of 103,167 angiographies were recorded, of which around 64,000 were coronary angiographies (CA). In other words, around 7 CA were performed per 1,000 inhabitants. The effective dose across all angiographies was 0.08 mSv per capita. Regarding the therapeutic interventions, a total examination frequency of 113,739 was calculated, which corresponds to a frequency of 12.7 per 1,000 inhabitants. Percutaneous coronary interventions (PCI) accounted for 30% of those, which made up almost 69% of the dose. The effective dose is 0.12 mSv per capita.

**Table 3.** The table shows the effective dose, collective effective dose, frequency per 1,000 inhabitants, and dose per capita for diagnostic and therapeutic interventional procedures.

Anatomical region	E per examination (mSv)	S (person-Sv)	Frequency of examinations per 1,000 inhabitants	E per capita (mSv)
Interventional radiology - diagnostic procedures				
Coronarography	7	49.7	7.1	0.050
Angiography (abdominal/pulmonary/pelvic/cerebral/other)	9.2	26.68	2.9	0.026
Angiography (lower extremities)	3.1	2.48	0.8	0.002
Angiography (right heart)	1.1	0.44	0.4	0.0005
Angiography (upper extremities)	0.11	0.033	0.3	0.0000
Interventional radiology - therapeutic procedures				
Percutaneous coronary interventions	20.6	78.28	3.8	0.079
Therapeutic interventions (various)	5	29.5	5.9	0.030
Electrophysiology	2.6	3.12	1.2	0.003
Pacemaker/Defibrillator	2.65	2.65	1	0.003
Heart valve	1.9	0.38	0.2	0.0004
Pain management	0.5	0.25	0.5	0.0003

Sex and age distribution was possible for radiology. For example, male patients undergo more thorax and abdominal CT scans than female patients, but the number of neurocranial scans is approximately the same for both sexes. While neurocranial scans are performed to pediatric patients (0 to 10 years old), thorax and abdomen CT are less frequent for such young patients. For adults aged up to 80, neurocranial CT examinations are performed half as often as thorax and abdomen CT examinations. For older patients, however, the frequency of all three examinations is equal, and for patients over 90, neurocranial CT examinations are twice as frequent as the other two. Male patients underwent twice as many CA and PCI procedures as female patients. For all patient age categories, the number of CA procedures is twice the number of PCI procedures. Regarding mammography, screening programs start at the age of 50 y, meaning that most of the examinations are performed to women between 50 and 70 y. Few women need diagnostic mammography before the age of 40 y, and most of them are between 50 and 80 y when they need a diagnostic examination.

### Dental examinations

The dental survey achieved a high overall response rate to the online survey of approximately 74%, with cantonal response rates ranging from 50% to 100%. This high response rate ensured good representativeness of the derived frequencies for dental examinations. In total, just over 6 million dental examinations were carried out in 2023. This equates to 671 examinations per 1,000 inhabitants (Table 4). Almost 80% of these were intraoral x rays. Cone Beam CT scans accounted for only 3.4% of the frequency but contributed 29% to radiation exposure. The collective effective dose per capita in dentistry was around 0.01 mSv.

**Table 4.** The table shows the effective dose, collective effective dose, frequency per 1,000 inhabitants, and dose per capita for dental examinations.

Dental examination	E per examination (mSv)	S (person-Sv)	Frequency of examinations per 1,000 inhabitants	E per capita (mSv)
Intraoral radiography	0.01	47.7	532.6	0.005
Panoramic radiograph/ orthopantomogram	0.02	16.1	89.6	0.002
Cephalometry	0.01	2.4	26.2	0.0003
CBCT	0.13	26.4	22.7	0.003

### Nuclear medicine

In nuclear medicine, a total of 140,397 examinations were performed in 2023, of which 83,658 were PET and 56,739 were SPECT scans. This equates to 15.7 examinations per 1,000 inhabitants. The effective dose from nuclear medicine examinations was 0.11 mSv per capita, with the CT part accounting for 47.7% of the total dose. PET tumor examinations accounted for over 50% of the frequency and almost 75% of the collective effective dose. The results for PET/CT and SPECT/CT examinations, differentiated by protocols and radiopharmaceutical/CT contributions, are presented in Table 5.

**Table 5.** The table shows the effective dose, collective effective dose, frequency per 1,000 inhabitants and dose per capita for PET/CT and SPECT/scintigraphy, differentiated by protocols and radiopharmaceutical/CT contributions.

Protocol	E (mSv) Radiopharma-ceutical part	E (mSv)-CT part	S (person-Sv)	Frequency of examinations per 1,000 inhabitants	E per capita (mSv) Radiopharma-ceutical part	E per capita (mSv) CT part	E per capita (mSv)-Total
**PET/CT**							
Tumor	4.29	5.92	82.0	8.03	0.034	0.0475	0.082
Myocardium	1.06	0.56	2.1	1.27	0.001	0.0007	0.002
Brain	5.32	0.25	0.2	0.03	0.0002	0.00001	0.0002
**SPECT/CT - Scintigraphy**							
Skeleton	3.64	3.26	17.9	2.59	0.009	0.004	0.013
Myocardium	5.97	N/A	9.3	1.55	0.009	N/A	0.009
Thyroid	6.13	1.04	1.5	0.21	0.001	0.0001	0.0014
Brain	5.32	N/A	0.6	0.12	0.001	N/A	0.001
Pulmonary perfusion	1.05	1.28	1.1	0.47	0.0005	0.0003	0.0008
Kidneys	0.6	N/A	0.3	0.47	0.0003	N/A	0.0003
Lymphscintigraphy	0.08	N/A	0.07	0.86	0.0001	N/A	0.0001
Pulmonary ventilation	0.28	1.28	0.08	0.05	0.00001	0.00003	0.00004
Blood source	0.25	N/A	0.0008	0.003	0.000001	N/A	0.000001

## DISCUSSION

The change in the survey methodology from the previous years is of great significance. It is important to distinguish the approach taken here from the previous monitoring studies. The survey in 2008 was based on an online collection of data and a scalable projection based on different healthcare provider groups to cover the missing data, which led to an inaccuracy due to differences in the individual clinical practice (Samara et al. 2012). The surveys in 2013 and 2018 were based on raw data extracted directly from some hospital IT-systems (Bize et al. 2018; Le Coultre et al. 2013). This means that all data were available at the individual level, which allowed that individual examinations were mapped more accurately and there were fewer risks that tariff or treatment codes were “optimized” for billing purposes. This survey, however, was based on aggregated administrative and billing data and therefore suffered from a certain degree of inaccuracy. However, the downside of the previous approaches was that data were hardly scalable to a national level. In this survey, no extrapolation was needed because the billing and administrative data cover the whole country. Thanks to the large sample size in dentistry, results are very robust for this specialty. Table 6 summarizes the development of the frequency and dose per capita since 2008. In the column “Comments” of Table 6, further differences among the surveys were stated, such as the estimation of E per procedure and the inclusion of nuclear medicine examinations.

**Table 6.** Comparison of frequency and dose data in 2023 with those of previous surveys.

Survey year	Frequency per 1,000 population	E per capita (mSv)	Comments
2008	1,700	1.2	E estimation, without NM examinations, survey to all healthcare providers, projection uncertainties
2013	1,219	1.4	E from 2008, without NM examinations, data from one hospital, projection uncertainties
2018	1,229	1.5	E from 2008, with NM examinations, data from a few hospitals, projection uncertainties
2023	1,443	1.7	E estimation, with NM examinations, billing and administrative data covering all healthcare providers, no projection, uncertainties in billing interpretations

**Fig. 3:**
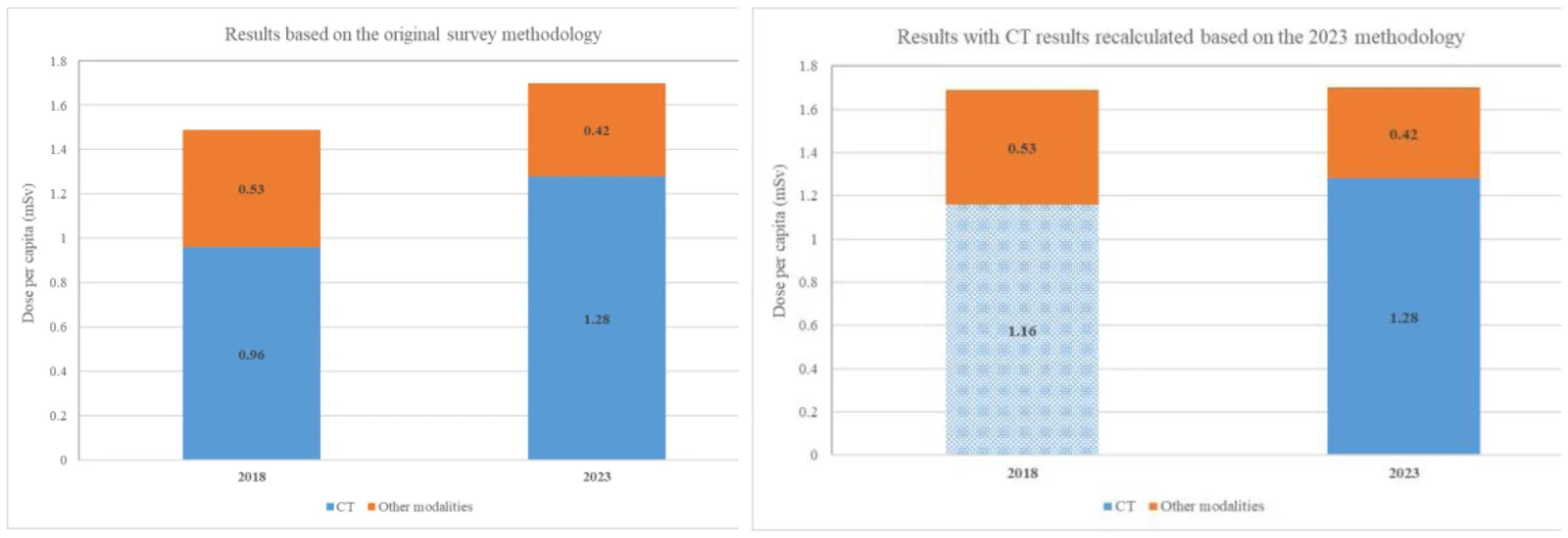
Evolution of collective effective dose between 2018 and 2023. The results from the original surveys are presented on the left, while the frequencies for CT in 2018 were recalculated following the 2023 methodology (right).

The effective dose in this survey was estimated at 1.69 mSv per capita, representing an apparent increase of 13% compared with 2018 (Viry et al. 2021). The primary reason for this was the increase in the number of CT scans. While 135 CT scans per 1,000 inhabitants were recorded in 2018, this figure had risen to 207 by 2023. Thus, compared to 2018, there has been a 33% increase in the collective effective dose for CT scans. Applying the 2023 method retroactively to the 2018 survey showed that the actual radiation exposure in 2018 was already at today’s level. The effective dose per capita for CT changes to 1.16 mSv (instead of 0.96 mSv with the previous method), while the effective dose of the other modalities remained unchanged. Fig. 3 illustrates the differences between the 2018 and 2024 surveys and demonstrates not only the importance of the chosen methodology but also the impact in the results.

Regarding conventional radiography, compared to 2018, the collective effective dose has decreased from 0.14 mSv to 0.04 mSv (Viry et al. 2021). This is almost entirely due to lower doses per examination.^1^ The same was observed for fluoroscopy. In 2018, the collective effective dose was 0.044 mSv per capita, while in 2023 0.03 mSv, representing a decrease of 39% (Viry et al. 2021). The decrease was exclusively due to lower doses per examination, while the frequency has increased from 5.5 to 12.2 examinations per 1,000 population.

The comparison with the 2018 survey showed that the frequencies for diagnostic interventional procedures were similar [9.9 (Viry et al. 2021) and 11.5 procedures per 1,000 population] but that radiation exposure has fallen by around 32% from 0.12 to 0.08 mSv due to lower doses per examination. For therapeutic interventional procedures, the comparison with 2018 showed similar values, particularly for PCI (Viry et al. 2021). However, this survey reported higher frequencies for the remaining therapeutic procedures, which was due to the number of tariff codes analyzed following the new methodology.

Compared to 2018 (0.012 mSv) (Viry et al. 2021), a 150% increase in radiation exposure related to mammography examinations was observed. There are three main reasons for this increase: (1) higher frequency of examinations [an increase in the frequency of examinations is entirely plausible due to greater awareness among the population and the numerous cantonal programs promoting early detection (screening) of breast cancer], (2) different methodology followed between 2018 and 2023, and (3) higher dose per protocol.^1^

The collective effective dose per capita in dental x rays in 2023 decreased by 23% since 2018 (Viry et al. 2021). The decline was primarily due to lower doses per image for intraoral x rays and digital volume tomography. In dentistry, two-dimensional images are considered to emit a very low dose of radiation. Digitization has further reduced these already low doses. In addition to imaging modalities that reduce the dose, digital technology offers the option of selecting higher-resolution, and thus higher-dose, images. Thus, two opposing trends can be observed in dentistry. On the one hand, all dental x rays can be taken with an increasingly low dose per image. However, the frequency of imaging appears to be steadily increasing, which negates this effect. The growing use of higher-dose imaging techniques, particularly DVT, reinforces this trend.

A comparison with 2018 showed that the frequency of nuclear medicine examinations has increased slightly, but the radiation exposure from radiopharmaceuticals fell by around 10%. Overall, radiation exposure in nuclear medicine has increased by just under 3%. The reason for this is the increased number of PET examinations (+53% compared to 2018) accompanied by CT (Viry et al. 2021).

The extreme cases of dental x rays and CT scans make particularly evident how important the E per procedure is: dental x rays accounted for 47% of all examinations in 2023 but contributed only 0.6% to the dose — hence, linked to very low patient exposure per examination. The opposite was true for computed tomography: the frequency share was only 14.4%, but the contribution to total exposure was 76%. Diagnostic and therapeutic interventional procedures and PET/CT examinations also contributed to the collective effective dose, although their contribution to the frequency of examinations was in total 2.3%. This shows that these procedures are related to particularly high E per procedure.

The survey has its limitations. Besides the CT evolution, the evolution of the interventional procedures had also to be addressed. As previously mentioned, more interventional procedures could be considered in this survey than in the previous ones.

The collective category for “other examinations” included angioplasty on other blood vessels, embolizations, as well as gastrointestinal tract and urogenital interventions, which may significantly differ in terms of radiation dose. Procedures performed in operating rooms, such as orthopedic surgery, vascular surgery, etc., were not recorded as the codes were either too generic so the procedure could not be attributed to the correct category or there was a risk of double counting the frequency in case of combination of procedures. This represents a limitation, as on the one hand the use of x rays outside the radiology department is common, and on the other hand the radiation dose for certain procedures is particularly high due to their complexity. Therefore, interventional procedures should be more carefully evaluated in future surveys.

Table 7 compares the dose per capita obtained in the current study with the data reported recently by other countries: Austria, France, Germany, Greece, the United States of America, and the UNSCEAR estimation for high-income level countries that covers the decade 2008-2018. The collective effective dose per capita of 1.69 mSv in Switzerland compares well with those reported in other countries as well as the UNSCEAR calculations. Differences in the methodology of each national survey, such as accounting for dental examinations, including nuclear examinations (both nuclear and CT part or only CT part), covering all examinations or the most frequent ones, etc. may explain any differences.

**Table 7.** Comparison of the Swiss dose data in 2023 with other national and international surveys.

Country	Year	E/capita (mSv)	Comments	Reference
Switzerland	2023	1.69	E estimation, with NM examinations, billing and administrative data covering all healthcare providers, no projection, uncertainties in billing interpretations	This study
France	2022	1.57	E estimation, sample extraction from the National Health Data System, including, projection for population	(ASNR 2022)
Germany	2021	1.4	Billing and administrative data covering all healthcare providers, no projection	(Strahlenschutz 2021)
USA	2019	2.2	Billing and administrative data covering all healthcare providers, no projection, uncertainties from different systems	(NCRP 2019)
UNSCEAR	2018	1.7	E estimation, with NM examinations, national data, projection by healthcare/income level	(UNSCEAR 2022)
Greece	2018	1.8	E and frequency estimation (for certain examinations), projection according to the number of laboratories	(Askounis et al. 2018)
Austria	2015	1.5	Top20, billing and administrative data covering all healthcare providers, no projection	(Wachabauer et al. 2020)

## CONCLUSION

The current survey of 2023 estimated the frequency and dose distribution for all radiological modalities, including nuclear medicine, for the Swiss population. The annual dose per capita was estimated at 1.69 mSv. The survey was based on a methodologically robust combination of billing data, administrative data, and supplementary primary data collection. This enabled country-wide and largely automated recording of examination frequencies and dose values, while deliberately addressing conflicts between data availability, accuracy, and scalability. The methodological break with previous monitoring was explained and justified. The monitoring of the population dose provides a reliable basis for decision-making in health policy and radiation protection.

## Footnotes

9 Samara ET, Ott B, Dagassan-Berndt D, Effective dose estimations for the Swiss survey on medical radiation exposure of the population in 2023, Health Physics (accepted for publication).
